# Metabolite‐based genome‐wide association study enables dissection of the flavonoid decoration pathway of wheat kernels

**DOI:** 10.1111/pbi.13335

**Published:** 2020-03-21

**Authors:** Jie Chen, Xin Hu, Taotao Shi, Huanran Yin, Dongfa Sun, Yuanfeng Hao, Xianchun Xia, Jie Luo, Alisdair R. Fernie, Zhonghu He, Wei Chen

**Affiliations:** ^1^ National Key Laboratory of Crop Genetic Improvement and National Center of Plant Gene Research (Wuhan) Huazhong Agricultural University Wuhan China; ^2^ College of Plant Science and Technology Huazhong Agricultural University Wuhan China; ^3^ National Wheat Improvement Center Institute of Crop Sciences Chinese Academy of Agricultural Sciences Beijing China; ^4^ Max‐Planck‐Institute of Molecular Plant Physiology Potsdam‐Golm Germany

**Keywords:** flavonoid decoration, metabolite‐based genome‐wide association study (mGWAS), metabolomics‐associated breeding, pathway elucidation, wheat kernels

## Abstract

The marriage of metabolomic approaches with genetic design has proven a powerful tool in dissecting diversity in the metabolome and has additionally enhanced our understanding of complex traits. That said, such studies have rarely been carried out in wheat. In this study, we detected 805 metabolites from wheat kernels and profiled their relative contents among 182 wheat accessions, conducting a metabolite‐based genome‐wide association study (mGWAS) utilizing 14 646 previously described polymorphic SNP markers. A total of 1098 mGWAS associations were detected with large effects, within which 26 candidate genes were tentatively designated for 42 loci. Enzymatic assay of two candidates indicated they could catalyse glucosylation and subsequent malonylation of various flavonoids and thereby the major flavonoid decoration pathway of wheat kernel was dissected. Moreover, numerous high‐confidence genes associated with metabolite contents have been provided, as well as more subdivided metabolite networks which are yet to be explored within our data. These combined efforts presented the first step towards realizing metabolomics‐associated breeding of wheat.

## Introduction

Plant metabolites play crucial roles in the interaction of plants with their surrounding environments (Saito and Matsuda, 2010; Schwab, 2003) and are necessary for humans in that they directly or indirectly constitute our nutritional supply (De Luca *et al.*, 2012; Keurentjes, 2009; Saito and Matsuda, 2010; Wang *et al.*, 2009). One class of specialized metabolites (also called secondary metabolites), the flavonoids, have been proposed to possess a range of functional roles. For instance, different decorations of the basic flavonoid structure were associated with varied UV tolerance in both rice cultivars and *Arabidopsis* ecotypes dispersed in various latitudes (Peng *et al.*, 2017; Tohge *et al.*, 2016), and flavonoid metabolites are believed to confer, among other bioactivities, anti‐inflammatory activity when provided in the diet (Kang *et al.*, 2016; Martin and Li, 2017; Zhou and Ibrahim, 2009). However, the enormous number of predicted metabolites (Dixon and Strack, 2003) and the severe variation in their abundance between species (Morohashi *et al.*, 2012) mean that vast majority of metabolic pathways remain to be fully unveiled. Indeed, unlike primary metabolites which are similarly present across the plant kingdom, secondary metabolic pathways are highly divergent between species. An elegant recent study revealed that part of the previously known pathway for flavonoid syntheses was reconstructed in rice, in which the naringenin‐to‐tricin route was redirected bypassing the formation of tricetin (Lam *et al.*, 2015). More recently, the progress in flavonoid biosynthesis pathways in model plants and several crop species has been reviewed (Tohge *et al.*, 2017). These studies demonstrated the essentiality of applying metabolomics as a systematic approach to study specialized plant metabolism. Specifically, metabolomic genome‐wide association study (mGWAS) or metabolomic quantitative trait loci mapping (mQTL) has proven highly powerful in understanding the diversification of metabolites (Chen *et al.*, 2014; Gong *et al.*, 2013; Zhu *et al.*, 2018), as well as the association of these metabolites with biotic and abiotic stress defence processes (Chen *et al.*, 2018; Glauser *et al.*, 2011; Peng *et al.*, 2017) or with food quality and flavour (Peng *et al.*, 2016; Sharma *et al.*, 2016; Tieman *et al.*, 2017). However, to date only very few mGWAS or mQTL studies have been conducted in wheat (Hill *et al.*, 2013; Hill *et al.*, 2015; Matros *et al.*, 2017).

Wheat (*Triticum aestivum* L.) is a leading cereal crop ultimately accounting for approximately 20% of the calories consumed by humans (Simmonds *et al.*, 2016). To secure worldwide food supply, intense selection of high‐yield and broad‐adaptation wheat cultivars has been the primary breeding target in wheat breeding programmes (Dubcovsky and Dvorak, 2007). However, owing to linkage drag and relatively low recombination frequencies, conventional breeding processes are usually time‐consuming and have low efficiency and predictability (Holland, 2007), which renders it necessary to develop and incorporate genomic tools to assist wheat breeding programmes (Simmonds *et al.*, 2016). Indeed, the available genomic information of hexaploid wheat (IWGSC, 2018) and its tetraploid (Avni *et al.*, 2017) and diploid progenitors (Ling *et al.*, 2018; Luo *et al.*, 2017) has greatly facilitated fundamental research in wheat (Cui *et al.*, 2019; Ju *et al.*, 2019) and may ultimately aid in resolving the above‐mentioned field problems of this crop.

Here, a mGWAS study was conducted on a collection of 182 wheat accessions, with a 90K SNP chip being used for genotyping. A total of 805 metabolites were detected from wheat grains, and 14 646 polymorphic SNPs were subjected to the association study. Consequently, 1098 marker–metabolite associations were detected, and 26 candidate genes for 42 of these loci were tentatively assigned. By enzymatically validating these candidates and testing their specificities on different flavonoid metabolites as substrates, a major flavonoid decoration pathway was representatively unravelled in wheat. By providing numerous high‐confidence candidate genes and suggesting the capacity for future metabolic pathway elucidation is resident within our data, we propose greater efforts are required towards realizing the full potential of metabolomics‐associated breeding in wheat.

## Results

### Metabolic profiling of wheat mature seeds

**Figure 1:**
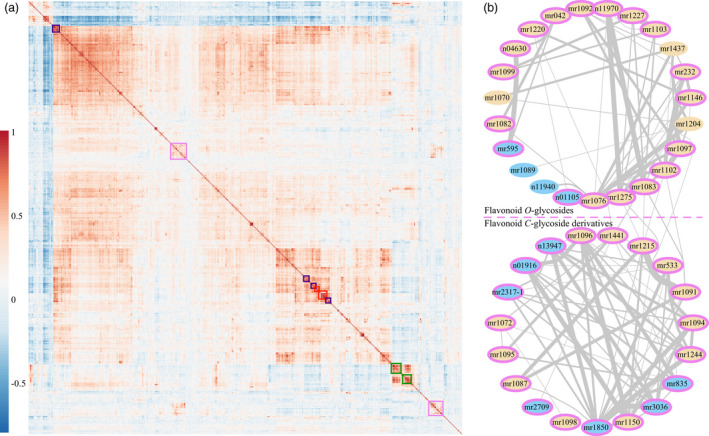
Correlation analyses of metabolites. (a) The metabolites were clustered based on their distribution patterns among the 182 wheat accessions; the red‐to‐blue colours for each cell represent the two corresponding metabolites sharing high‐to‐low similarly distributed patterns (indicated by the coefficient values) among wheat accessions. The coloured rectangles including metabolites are displayed as respectively colour‐ringed IDs in Figue 1b and Figures S3–S5. (b) Networks for flavonoid derivatives (violet‐backgrounded in Figure S3a), in which the identified metabolites appeared to be flavonoid glycosides (wheat‐coloured cells). Violet‐ringed IDs correspond to the violet rectangles including metabolites in Figure 1a. Lines indicated the linking metabolites shared similar distribution patterns among the 182 wheat accessions, wherein the thicker lines represented higher coefficient values. IDs for metabolites were listed in Table S1.

Using a previously established widely targeted metabolomics method (Chen *et al.*, 2013), a total of 805 metabolites (including 387 known and 418 unknown metabolites, Table S1) from mature seeds of 182 wheat accessions were detected (Table S2). The relative contents for each metabolite from respective environments were normalized (log_2_‐transformed, Figure S1) before being subjected to downstream data analyses. These metabolites exhibited varied broad‐sense heritability (Figure S2a) and normally distributed coefficients of variation (Figure S2b) among the 182 wheat accessions. Correlation analysis of the distribution patterns for the 805 detected metabolites was displayed (Figure 1a), in which the blocks along the diagonal (represented by the coloured rectangles in Figure 1a) suggested the distribution patterns of the included metabolites were highly correlated with one another, and they may share similar chemical structure or be involved in related metabolic pathways. Specifically, the flavonoid derivatives (wheat‐coloured cells in Figure S3) could form a relatively isolated sub‐net and several sparsely linked ties with cut‐off coefficient indexes over 0.55 (Figure S3). Further inquiry on this designated sub‐net (IDs included in the violet‐backgrounded area, Figure S3a) suggested most of the metabolites (violet‐ringed IDs in Figure 1b) were situated alongside the diagonal in the heatmap (violet rectangles in Figure 1a), and the included flavonoid glycosides could be roughly separated into two sub‐groups regarding the presence or absence of *C*‐glycosyl moieties (Figure 1b). Meanwhile, another sub‐net (indicated by the green dashed circle in Figure S3), which could be easily discerned under a higher cut‐off coefficient index over 0.60 (the green dashed circle in Figure S4a), was discernible. Metabolites within this sub‐net may be involved in the metabolic pathways related to indole ring formation, decoration or degradation (or the tryptophan metabolism pathway). The reason for this assumption is that most of these metabolites (26 of 32, the green‐ringed IDs in Figure S4b) shared highly similar distribution patterns (indicated by the green rectangles in Figure 1a), and indole ring skeletons (green‐coloured part for each chemical structure in Figure S4b) were commonly found within the currently identified metabolites. Similarly, the vitamin sub‐net and nucleotide derivatives (sub‐nets 3 and 4 in indigo and scarlet colours, respectively, in Figure S5) were uncovered when a coefficient value threshold of over 0.75 was set, and these followed the diagonal‐distribution patterns (the indigo and scarlet rectangles, respectively, in Figure 1a) as stated above.

### Metabolite genome‐wide association study (mGWAS)

**Figure 2:**
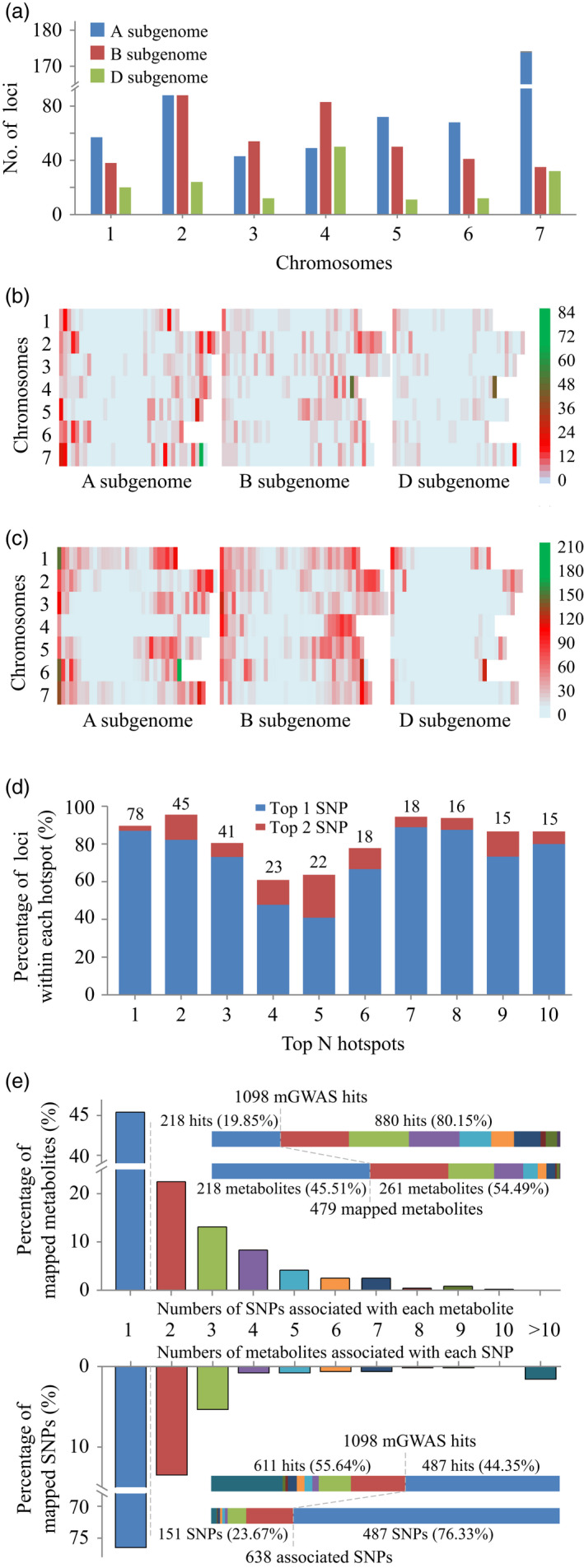
Statistical analyses of mGWAS associations. (a) Distribution of 1098 mGWAS hits among the 21 wheat chromosomes. (b) Location of these mGWAS hits within the continuous 20‐Mb intervals on each chromosome. (c) Attribution of the 14 646 polymorphic SNPs within the continuous 20‐Mb intervals on each chromosome. (d) Numbers of mGWAS hits within each hotspot are labelled at the top of respective columns, these mGWAS loci were largely associated with a few SNPs. (e) The 1098 mGWAS hits were differentially attributed by SNPs or metabolites. The grey dashed lines indicated percentages of SNPs (metabolites) contribute to respective proportion of mGWAS hits.

Following the evaluation of the metabolite profiling data, we next performed mGWAS analyses based on the 14 646 polymorphic SNPs (by eliminating those with minor allele frequencies (MAFs) <0.05, or missing data >20%, Table S3) from the wheat 90K Illumina iSelect SNP Array (Wang *et al.*, 2014). A total of 1098 SNP–metabolite associations (Table S4) were detected under the threshold of 6.83 × 10^−5^ after Bonferroni correction. These mGWAS hits were distributed preferentially on the A and B subgenomes rather than on the D subgenome (Figure 2a), and they were located at distal chromosomal regions over the 21 wheat chromosomes (Figure 2b). The top‐three hotspots located on the 700–720 Mb region of chromosome 7A, 640–660 Mb region of chromosome 4B and 500–520 Mb region of chromosome 4D, comprising 77, 45 and 41 loci, respectively (Figure 2b), which covered 44.25%, 54.22% and 82.00% of identified associations on their respective chromosomes. Similar to the overall mGWAS hit distribution, the SNP markers were preferentially located on the A and B subgenomes rather than the D subgenome, and they mapped more frequently at the distal chromosomal regions (Figure 2c). The overall consistency between the mGWAS allocation (Figure 2b) and SNP attribution (Figure 2c) led us to question if there is a connection between loci frequency and marker density. Opposite to this intuition, most of the associations for each of the top‐three hotspots were attributed to single SNPs: 67 of 77 (87.01%) associations from the 700 to 720 Mb region of chromosome 7A were linked to SNP *BS00022811_51* located at 709.64 Mb, most of the loci (37 of 45, 82.22%) within the 640‐660 Mb region of chromosome 4B were associated with SNP *Excalibur_c29255_366* at 657.47 Mb, and the hotspot on the 500‐520 Mb region of chromosome 4D was mainly (30 of 41 loci, 73.17%) associated with SNP *Kukri_c49387_1187* at 509.83 Mb (Table S5). This situation was similarly held for the top‐ten mGWAS hotspots in that the involved associations within each hotspot were largely mapped to one or two SNPs (Figure 2d and Table S5). Overall, mono‐mGWAS hits were associated with nearly half of the mapped metabolites (218 of 479) or three‐quarters of the mapped SNPs (487 of 638, Figure 2e). That is, 880 (80.15%) and 611 (55.64%) of the 1098 metabolite–SNP associations were attributed to 261 (54.49% of 479) and 151 (23.67% of 638) of the mapped metabolites and SNPs, respectively (Figure 2e), which confirmed the postulation that a large number of associations were generated from a relatively low proportion of SNPs or metabolites.

### Identification and functional annotation of candidate genes underlying mGWAS associations

After analysing the attribution of the mGWAS associations, we next went on to probe candidate genes underlying these metabolite–SNP associations. In most cases, the chemical structure of the metabolites, existing knowledge of the pathway architecture and the gene features within the linkage disequilibrium (LD) coverages (Table S6) allowed the tentative assignment of a protein or protein cluster as regulating the metabolic traits (Chen *et al.*, 2014). Using this knowledge allowed possible candidate genes to be assigned. For example, we tentatively assigned the candidate genes *TraesCS4B01G371700* (657.17 Mb on chromosome 4B) and *TraesCS4D01G365800* (509.66 Mb on chromosome 4D), within the LD range (3.31 Mb and 1.12 Mb for chromosomes 4B and 4D, respectively, Table S6), for the mr373 (sucrose) associated with *Excalibur_c29255_366* (657.47 Mb on chromosome 4B) and *Kukri_c49387_1187* (509.83 Mb on chromosome 4D), respectively. The top hit of these candidates (*TraesCS4B01G371700* and *TraesCS4D01G365800*) in *Arabidopsis* (*AT3G19940*, also known as *SPT10*) encodes a high‐affinity hexose transporter carrying glucose and other monosaccharides (Paulsen *et al.*, 2019; Rottmann *et al.*, 2016). Similarly, for the mGWAS hit (*P* = 1.21 × 10^−8^) of metabolite mr1013 (identified as *N*‐feruloylagmatine) with SNP *RAC875_c47743_81* at 712.83 Mb on chromosome 2B, the nearby candidate was putatively designated as *TraesCS2B01G518300* (Table 1 and Table S4). This candidate was annotated as ‘agmatine coumaroyl‐transferase’ and shared high sequence similarity with rice gene *LOC_Os04g56910* (75.84% amino acid identity), which was previously functionally validated *in vivo* as an agmatine hydroxycinnamoyl acyltransferase (Chen *et al.*, 2014).

**Table 1:** Candidate genes assigned in the current study

Metabolite	Lead SNP	*P*‐value	Candidate gene	Description^a^	Distance (Mb)^b^
*C*‐pentosyl‐apeignin *O*‐feruloylhexoside	RFL_Contig5637_1008	8.85E‐12	TraesCS1A01G021100	Transferase	0.57
*C*‐pentosyl‐apeignin *O*‐feruloylhexoside	GENE‐3569_500	9.61E‐06	TraesCS1A01G032300	CHS	0.59
*C*‐pentosyl‐apeignin *O*‐feruloylhexoside	Tdurum_contig61410_467	6.68E‐06	TraesCS1A01G037800	PAL	0.04
Tricin	BS00028146_51	9.78E‐07	TraesCS1A01G347100	Glycosyltransferase	0.19
Tricin 7‐*O*‐hexosyl‐*O*‐xyloside	Kukri_c25138_1155	5.84E‐15	TraesCS1B01G335900	Glycosyltransferase	0.11
*C*‐pentosyl‐apeignin *O*‐feruloylhexoside	Excalibur_c46114_728	1.91E‐06	TraesCS1D01G020700	Transferase	0.13
Tricin 7‐*O*‐hexosyl‐*O*‐xyloside	RAC875_c49612_102	1.60E‐21	TraesCS1D01G319100	Glycosyltransferase	1.21
Tricin *O*‐malonylhexoside	BobWhite_c1933_407	1.41E‐07	TraesCS2A01G450700	Transferase	1.20
3,4‐Dihydroxybenzaldehyde	wsnp_Ex_rep_c69692_68647924	6.32E‐07	TraesCS2A01G468200	Polyphenol oxidase	0.39
Tricin *O*‐malonylhexoside	Tdurum_contig5691_596	8.18E‐08	TraesCS2B01G472400	Transferase	0.57
3,4‐Dihydroxybenzaldehyde	GENE‐0808_728	9.40E‐06	TraesCS2B01G491100	Polyphenol oxidase	0.89
*N*‐Feruloylagmatine	RAC875_c47743_81	1.21E‐08	TraesCS2B01G518300	Transferase	0.06
*C*‐hexosyl‐chrysoeriol 5‐*O*‐hexoside	D_contig21303_418	1.04E‐08	TraesCS2D01G043500	Cytochrome P450	0.11
Tricin	RAC875_c17404_1160	6.22E‐05	TraesCS3A01G226600	DFR	0.01
Lysine	RAC875_c1022_3059	4.67E‐06	TraesCS4A01G294100	Aminopeptidase	0.42
Sucrose	Excalibur_c29255_366	7.92E‐07	TraesCS4B01G371700	Sugar transporter	0.30
Caffeic acid	Kukri_c49387_1187	6.74E‐07	TraesCS4D01G362500	*O*‐methyltransferase	1.02
Sucrose	Kukri_c49387_1187	1.09E‐06	TraesCS4D01G365800	Sugar transporter	0.16
Oleamide	BobWhite_c14689_172	5.94E‐06	TraesCS5A01G433100	Lipid transferase	0.10
Phytocassane D	RAC875_c22599_731	1.63E‐06	TraesCS5A01G521600	Terpene synthase	0.04
3‐*O*‐Feruloylquinic acid	wsnp_Ku_c4299_7814936	2.53E‐07	TraesCS7A01G023800	Transferase	0.34
3′,4′,5′‐Tricetin *O*‐rutinoside	BS00084039_51	4.36E‐05	TraesCS7A01G041200	F3′H	0.30
Terephthalic acid	RAC875_c35727_269	1.63E‐14	TraesCS7A01G054500	Polyphenol oxidase	0.69
MGMG (18:2)	BobWhite_c40479_283	8.70E‐06	TraesCS7A01G100600	GDSL lipase	0.95
Leucine	wsnp_BQ160404A_Ta_1_1	6.91E‐06	TraesCS7A01G464900	Amino acid transporter	0.83
Arginine	BS00022811_51	3.06E‐06	TraesCS7A01G531000	Peptide transporter	0.17

a CHS, chalcone synthase; DFR, dihydroflavonol 4‐reductase; F3′H, flavonoid 3′ hydroxylase; PAL, phenylalanine ammonia‐lyase.

b Distance denotes the physical distance between the assigned candidates and respective lead SNPs.

**Figure 3:**
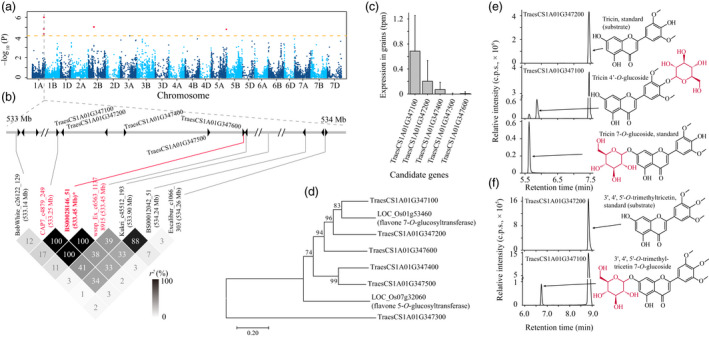
Validating that TraesCS1A01G347100 confers glucosyltransferase activity. (a) Manhattan plot displaying the mGWAS result of metabolite mr1058 (tricin), the significantly associated SNPs (above the threshold) are displayed as red dots. (b) A representation of pairwise *r^2^* value (displayed as percentages) among polymorphic sites of the mGWAS loci, in which the three SNPs above the threshold are labelled as red font, the lead SNP is indicated by an asterisk and displayed bold. Triangles indicate relative positions and directions of genes resided adjacent to the SNPs. Five adjacent glycosyltransferase‐coding genes (i.e. *TraesCS1A01G347100*, *TraesCS1A01G347200*, *TraesCS1A01G347400*, *TraesCS1A01G347500* and *TraesCS1A01G347600*) are designated as candidates. Based on the relative expression in wheat grains (c) and the sequence similarity to rice homologs (d), the enzymatic activities of TraesCS1A01G347100 and TraesCS1A01G347200 were tested, turned out only TraesCS1A01G347100 conferred expected glycosyltransferase activity on tricin as substrate (e). The early eluted product was confirmed as tricin 7‐*O*‐glucoside compared with commercial standard (e), and the other compound was postulated as tricin 4′‐*O*‐glucoside (e) which was supported by further enzymatic assay utilizing 3′, 4′, 5′‐*O*‐trimethyltricetin as substrate (f). The MS spectrums for the predicted products (tricin 4′‐*O*‐glucoside and 3′, 4′, 5′‐*O*‐trimethyltricetin 7‐*O*‐glucoside) are presented in Figure 5.

It is beyond the scope of a single study to experimentally check all the candidate genes involved, but we performed functional annotation via analysis of enzymatic activities in order to demonstrate the effectiveness of our candidate designating processes. For the associations between mr1058 (identified as tricin) and SNP *BS00028146_51* (*P* = 9.78 × 10^−7^) at 533.45 Mb on chromosome 1A (Figure 3a), five nearby glycosyltransferase‐coding genes were assumed as likely candidates (Figure 3b). Among them, we chose to express two genes (*TraesCS1A01G347100* and *TraesCS1A01G347200*) in *E. coli*, since these two candidates have relatively high expression levels in wheat grains (Figure 3c) and closer sequence similarities with the rice ortholog (Figure 3d) that confers flavone 7‐*O*‐glucosyltransferase function (Ko *et al.*, 2008; Peng *et al.*, 2016). It turned out that the former gene (i.e. *TraesCS1A01G347100*) was more likely the candidate for this mGWAS hit, the encoding product could catalyse the conversion of tricin to tricin 7‐*O*‐glucoside and tricin 4′‐*O*‐glucoside (Figure 3e), with *K*_m_ = 6.92 ± 0.86 μm and 6.49 ± 0.71 μm, and *K*_cat_ = 0.12 ± 9.72 × 10^−3^ s^−1^ and 0.64 ± 4.99 × 10^−2^ s^−1^, respectively.

**Figure 4:**
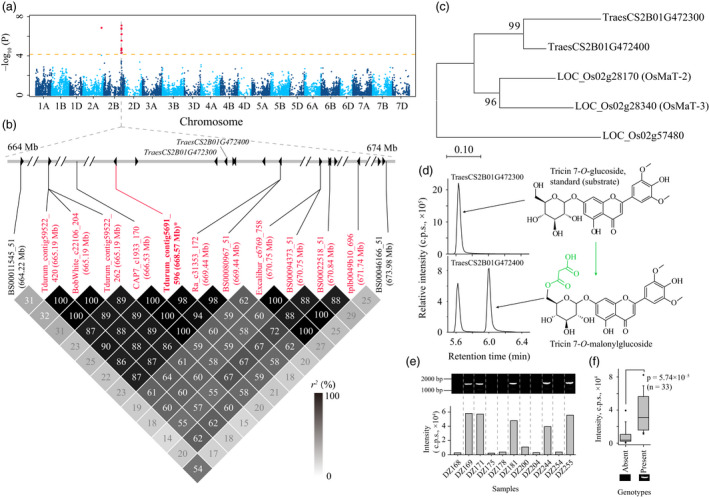
Validating that TraesCS2B01G472400 confers malonyl‐transferase activity. (a) Manhattan plot displaying the mGWAS result of metabolite mr1093 (tricin *O*‐malonylglycoside), the significantly associated SNPs (above the threshold) are displayed as red dots. (b) A representation of pairwise *r^2^* value (displayed as percentages) among polymorphic sites of the mGWAS loci, in which the eleven SNPs above the threshold are labelled as red font, the lead SNP is indicated by an asterisk and displayed bold. Triangles indicate relative positions and directions of genes resided adjacent to the SNPs. (c) Phylogenetic analysis of the two candidate genes with rice homologs (*OsMaT‐2* and *OsMaT‐3*). Another rice acyltransferase‐coding gene, *LOC_Os02g57480*, was applied as an outlier. (d) In vitro enzymatic assay of TraesCS2B01G472300 and TraesCS2B01G472400. The tricin 7‐*O*‐glucoside was used as substrates for both reactions, and tricin 7‐*O*‐malonylglucoside was generated as expected. The MS spectrum of the generated tricin 7‐*O*‐malonylglucoside is presented in Figure 5. (e) The consistence between *TraesCS2B01G472400* amplifying result with relative contents of mr1093 was presented in several wheat accessions, in which the absence/presence of PCR band corresponded to low/high contents of mapped metabolite (mr1093). (f) Statistical analysis of such correlation, wherein the samples were grouped based on the absence/presence (16/17 accessions) of amplification results, and *P*‐value was generated by Student's *t*‐test.

**Figure 5:**
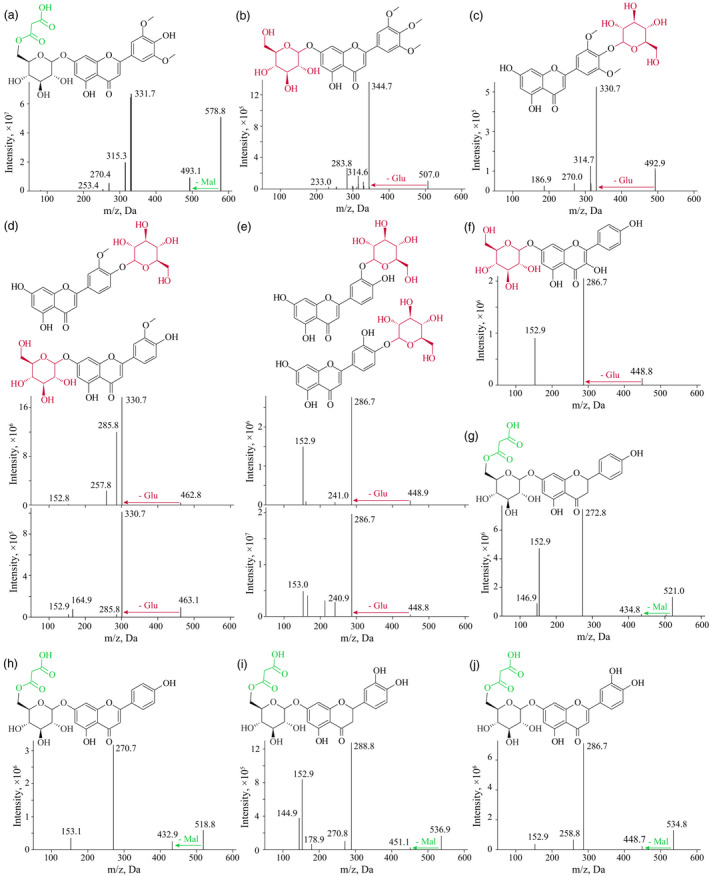
The MS spectrums and respective chemical structures of several products generated from the enzymatic assays. Information for tricin 7‐*O*‐malonylglucoside (a), 3′, 4′, 5′‐*O*‐trimethyltricetin 7‐*O*‐glucoside (b), tricin 4′‐*O*‐glucoside (c), chrysoeriol 4′‐*O*‐/7‐*O*‐glucosides (d), luteolin 3′‐*O*‐/4′‐*O*‐glucosides (e), kaempferol 7‐*O*‐glucoside (f), naringenin 7‐*O*‐malonylglucoside (g), apigenin 7‐*O*‐malonylglucoside (h), eriodictyol 7‐*O*‐malonylglucoside (i) and luteolin 7‐*O*‐malonylglucoside (j) are respectively presented.

As presented above, the mGWAS hits were largely associated with a few SNPs or metabolites, displayed as hotspots (Figure 2). On the SNP side, the responsible candidates may be retrieved by exploring the similarities among the simultaneously mapped metabolites. Indeed, SNP *D_contig21303_418* (15.52 Mb on chromosome 2D) was associated with numerous flavone derivatives (i.e. mr1099, mr1103 and mr1227, Table S4), and *TraesCS2D01G043500* (located at 15.63 Mb on chromosome 2D) was tentatively assigned as a candidate. Sequence alignment indicated this candidate shared high amino acid sequence similarity (73.81% identity) with the rice ortholog *CYP93G1*, which was responsible for the conversion of a flavanone (e.g. naringenin) to a flavone (e.g. apigenin) (Lam *et al.*, 2014). On the metabolite side, the associations between the same metabolites with SNPs located on the A, B and D subgenomes were probably due to the hexaploid nature of wheat, and homoeologous candidates may therefore be expected for these loci. Such an example was uncovered by the associations of metabolite mr1093 with SNPs *BobWhite_c1933_407* on chromosome 2A and *Tdurum_contig5691_596* on chromosome 2B (Table S4). For the metabolite mr1093 (putatively identified as tricin *O*‐malonylhexoside) with SNP *Tdurum_contig5691_596* (668.57 Mb on chromosome 2B) association (*P* = 8.18 × 10^−8^, Figure 4a), *TraesCS2B01G472300* and *TraesCS2B01G472400* (resided at 669.09 Mb and 669.14 Mb on chromosome 2B, respectively) were assigned as possible candidates (Figure 4b). Sequence alignment indicated the candidates' orthologs from rice, *OsMaT‐2* and *OsMaT‐3* (Figure 4c), were previously defined to encode malonyl‐transferase products (Gong *et al.*, 2013; Kim *et al.*, 2009). In confirmation, the enzymatic function for the two candidates was tested *in vitro*, with the results suggesting that only one of them, TraesCS2B01G472400, confers malonyl‐transferase activity on tricin 7‐*O*‐glucoside (Figures 4d and 5a). Survey on the enzymatic character of TraesCS2B01G472400 in catalysing the conversion of tricin 7‐*O*‐glucoside to tricin 7‐*O*‐malonylglucoside revealed the reaction constant as *K*_m_ = 20.53 ± 1.02 μm and *K*_cat_ = 81.72 ± 6.11 s^−1^. In addition, we encountered the presence/absence sequence variation when amplifying *TraesCS2B01G472400* (Figure 4e), and this pattern showed significant correlation with the variation in abundance of metabolite mr1093 (Figure 4f), suggesting the absence of a functional enzyme encoded by *TraesCS2B01G472400* is likely responsible for the lower contents of the product metabolite, tricin *O*‐malonylglucoside (i.e. mr1093). For mr1093 associated (*P* = 1.41 × 10^−7^) with the SNP *BobWhite_c1933_407* (698.83 Mb on chromosome 2A), *TraesCS2A01G450700* (700.02 Mb on chromosome 2A), a *TraesCS2B01G472400* homoeologue, was tentatively designated as a candidate. However, the enzymatic assay indicated this candidate did not possess malonylation activity on tricin 7‐*O*‐glucoside (data not shown), which may result from absent amino acids in the sequences when compared to TraesCS2B01G472400 in the Chinese Spring cultivar (Figure S6)*.* Taken together, these functional annotations (*TraesCS1A01G347100* for tricin and *TraesCS2B01G472400* for tricin *O*‐malonylglucoside) have demonstrated that our candidate gene assignment programme is effective, with enzymes encoded by respective candidates alternately being involved in the synthesis or degradation of the associated metabolites. In total, 26 candidate genes were tentatively designated for 42 mGWAS hits (Table S4).

### Pathway construction for flavonoid decoration

In the *in vitro* enzymatic validation of TraesCS1A01G347100, two peaks with different retention time (RT) were obtained (Figure 3e), which corresponded to two different tricin *O*‐glucosides. Tricin has three candidate sites at which it can be *O*‐glycosylated, and respective tricin *O*‐glycoside conjugates (tricin 5‐*O*‐glycoside, tricin 7‐*O*‐glycoside and tricin 4′‐*O*‐glycoside) may be obtained. Of the two products, the early eluting compound (at RT of 5.71 min) was confirmed to be the tricin 7‐*O*‐glucoside by comparison to the characteristics of the commercial standard (Figure 3e), whereas the identity of the later eluting peak (at RT of 5.90 min) is uncertain. After performing a similar enzymatic assay using 3′, 4′, 5′‐*O*‐trimethyltricetin as substrate, a sole glucosylated product (Figures 3f and 5b) was generated. This output, together with the knowledge that flavonoid 7‐*O*‐glycosylation and flavonoid 5‐*O*‐glycosylation enzymes generally differ at the amino acid sequence level (Figure 3d), and that the flavonoid 5‐*O*‐glucosides generally elute earlier than respective flavonoid 7‐*O*‐glucosides (Peng *et al.*, 2017), leads us to postulate that the unknown glucosylated products were likely to be tricin 4′‐*O*‐glucoside (Figures 3e and 5c) and 3′, 4′, 5′‐*O*‐trimethyltricetin‐7‐*O*‐glucoside (Figures 3f and 5b), respectively.

**Figure 6:**
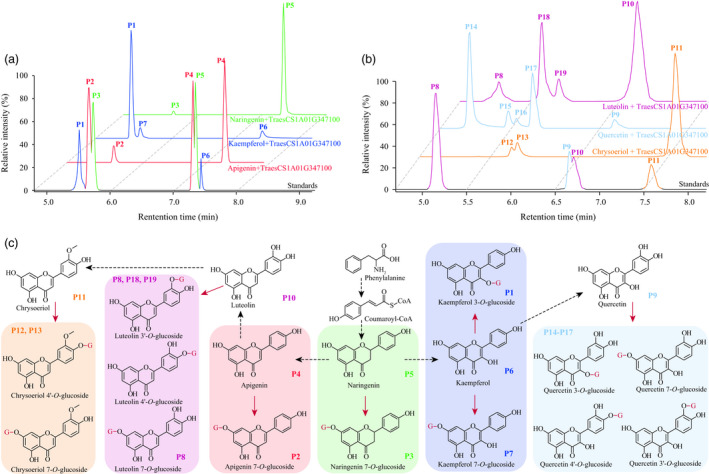
Substrate specificity of TraesCS1A01G347100. (a) TraesCS1A01G347100 could function on apigenin (a flavone), kaempferol (a flavonol) and naringenin (a flavanone), generating various glucosylated products. These flavonoids have single hydroxy group on the B‐ring. (b) Additional test of TraesCS1A01G347100 on chrysoeriol, luteolin and quercetin that have more hydroxy or methoxy groups on the B‐ring. (c) Illustration on how various flavonoids were glucosylated by TraesCS1A01G347100. This reaction was indicated by red arrows, and the glucosyl groups were represented by G in red. Peaks for each known metabolite were labelled on the right bottom of respective chemical structure, whereas MS spectrum for the postulated flavonoid glucosides is presented in Figure 5.

Considering previous reports on flavonoid glycosyltransferases (Ko *et al.*, 2006; Ko *et al.*, 2008), and the simultaneously generated tricin 7‐*O*‐glucoside and tricin 4′‐*O*‐glucoside as products from a single enzymatic assay (Figure 3e), we assume TraesCS1A01G347100 could function on various hydroxyl groups from numerous flavonoids. In confirmation, the enzymatic activities were tested using apigenin, kaempferol and naringenin as substrates, which turned out to respectively result in the production of apigenin 7‐*O*‐glucoside, two kaempferol *O*‐glucosides (kaempferol 3‐*O*‐glucoside and another kaempferol *O*‐glucoside) and naringenin 7‐*O*‐glucoside (Figure 6a). Again, the unknown kaempferol *O*‐glucoside (P7 in Figure 6a) was postulated to be kaempferol 7‐*O*‐glucoside for the above‐stated reason (Figure 3d), rather than kaempferol 4′‐*O*‐glucoside since neither apigenin 4′‐*O*‐glucoside nor naringenin 4′‐*O*‐glucoside were generated (Figure 6a). Collectively, TraesCS1A01G347100 appears to be capable of catalysing 7‐*O*‐glycosylation of flavones (e.g. apigenin) and flavanones (represented by naringenin), and 7‐*O*‐glycosylation or 3‐*O*‐glycosylation on flavonols (such as kaempferol, Figure 6c). This summary is not, however, entirely conclusive since both tricin 7‐*O*‐glucoside and tricin 4′‐*O*‐glucoside were generated via a single reaction (Figure 3e), whilst no apigenin/kaempferol/naringenin 4′‐*O*‐glucosides were obtained (Figure 6a). To probe whether the generation of tricin 4′‐*O*‐glucoside was a specific case when tricin is substrate or whether such a glycosylation product could represent a general product pattern when other flavonoids are used as substrates, additional tests of TraesCS1A01G347100 reaction products following supply of chrysoeriol and luteolin as substrates were conducted, in which two chrysoeriol glucosides and three luteolin glucosides (luteolin 7‐*O*‐glucoside and another two unknown luteolin glucosides) were obtained (Figure 6b). Combining the comparisons of chemical structures of the tested flavones (i.e. apigenin, chrysoeriol, luteolin, tricin and 3′, 4′, 5′‐*O*‐trimethyltricetin), for the respective enzyme activities, and substrate and product information (e.g. no flavonoid 5‐*O*‐glucoside conjugates were generated by TraesCS1A01G347100 catalysis), the two chrysoeriol glucosides were believed to be chrysoeriol 7‐*O*‐glucoside and chrysoeriol 4′‐*O*‐glucoside (Figure 5d), whilst the two unknown luteolin glucosides were assumed to be luteolin 3′‐*O*‐glucoside and luteolin 4′‐*O*‐glucoside (Figure 5e). Accordingly, the modification pattern was expanded such that the B‐ring *O*‐glycosylation from TraesCS1A01G347100 would not be expected in the instance that there is only one hydroxyl group attached to the B‐ring (Figure 6c). Consistently, four quercetin glucosides were obtained (Figure 6b), which were expected, although not respectively identified, to be quercetin 3‐*O*‐glucoside, quercetin 7‐*O*‐glucoside, quercetin 3′‐*O*‐glucoside and quercetin 4′‐*O*‐glucoside (Figure 6c).

Meanwhile, the possibility that differentially glycosylated flavonoids subsequently offer the substrate to form their respective *O*‐malonylglycoside conjugates was investigated. These studies revealed that the malonylation reaction catalysed by TraesCS2B01G472400 appeared to be specific for flavone/flavonol/flavanone 7‐*O*‐glycosides (Figure S7). Thus, as anticipated, these two enzymes (TraesCS1A01G347100 and TraesCS2B01G472400) could sequentially glycosylate and then malonylate designated flavonoids (Figure S8a) in a two‐step reaction (Figure S8b). Taken together, these data allowed us to establish the flavonoid decoration pathway as displayed in Figure S9.

## Discussion

The plant metabolome contains a vast variety of structurally different chemicals, termed metabolites, the contents of which are under considerable genetic control. It was concluded that metabolome variations within a species are much larger than that had been previously assumed (Fernie and Tohge, 2017). This renders the combination of metabolomics approaches and genetic tools such as QTL and GWAS (termed as mQTL and mGWAS, respectively) an effective path in exploring the genetic control of metabolome also facilitating the delineation of metabolic pathways and the dissection of agronomic traits (Chen *et al.*, 2018; Fang and Luo, 2019; Gong *et al.*, 2013; Shang *et al.*, 2014; Wen *et al.*, 2014; Zhu *et al.*, 2018). Such efforts had also been applied in wheat. Specifically, genetic elements affecting 205 compounds (within which 112 were identified, Hill *et al.*, 2013), 558 mass features (197 were identified, Hill *et al.*, 2015) or 76 metabolites (73 identified, Matros *et al.*, 2017) were investigated, respectively. That said, no candidate genes were provided (Hill *et al.*, 2013; Hill *et al.*, 2015) or validated (Matros *et al.*, 2017) within these wheat mQTL or mGWAS cases. In the current study, we investigated at a greater number of metabolites (805 metabolites, of which 387 were identified), and thanks to the Chinese Spring reference genome information (IWGSC, 2018), candidate genes for the mGWAS hits were identified (Table 1 and Table S4). Furthermore, enzymatic functions for some candidates were validated (Figures 3 and 4) via heterologous overexpression experiments. Finally, a flavonoid decoration pathway was elucidated (Figure S9) according to the enzymatic specificity tested on numerous substrates (Figures 6, S7 and S8). To the best of our knowledge, this work represents the first application of metabolomics for pathway elucidation in wheat, which may shed lights to unveil more metabolic pathways of wheat.

Metabolic pathways are constituted by highly diverse yet somewhat interconnected metabolites, which could be somehow classified as chemical decorations on several core structures (D'Auria and Gershenzon, 2005). Consistent with this theory, we could discriminate the flavonoid glycosides from the total detected metabolites by analysing the distribution patterns among wheat samples (Figures 1 and S3). Meanwhile, the similarly grouped tryptophan metabolism sub‐net (Figures 1a, S3a and S4), amino acids or nucleotide derivatives (Figures 1a and S5) also emerged when performing correlation analysis at different stringency of thresholds. Such groupings may facilitate the identification of currently unknown metabolites (Chen *et al.*, 2016), and future studies could thus focus on the genetic architecture of these individual small targeted classes of metabolites (Chan *et al.*, 2010). In *Arabidopsis*, only a mere 13 flavonoids were initially reported (D'Auria and Gershenzon, 2005). Following this, more than three times the number of flavonoid derivatives were detected (Nakabayashi *et al.*, 2009; Tohge and Fernie, 2010), and recently, another 18 metabolites – the saiginols – have been added into this pathway (Tohge *et al.*, 2016). Thanks to the continuous interest, the flavonoid metabolites have additionally been extensively studied in model plants and crop species (Tohge *et al.*, 2017). This research progress provides promising prospects in exploring the metabolomic profiling data presented here, since it indicates that it may improve our understanding of the subnetworks including flavonoid, tryptophan, amino acid or nucleotide metabolism. Such targeted studies will hopefully be up‐scaled to allow determination of the entire wheat metabolome.

The importance of exploring the realm of the metabolome is evident, given the widely accepted concept that this arsenal of compounds provides an effective means of defence against biotic and abiotic stresses, as well as contributing to the nutritional quality of designated plant species (Martin and Li, 2017; Rai *et al.*, 2017; Weng, 2014). Indeed, species‐specific metabolites conferring some of these bioactivities have attracted enormous interests, such as glucosinolates in the Brassicaceae (Grubb and Abel, 2006; Nour‐Eldin *et al.*, 2017), steroidal glycoalkaloids in the Solanaceae (Itkin *et al.*, 2013), isoflavones in leguminous species (Tohge *et al.*, 2017; Veitch, 2013) and cucurbitacins in cucurbits (Shang *et al.*, 2014). Such a specifically existed yet physiologically related metabolome route may also be explored in wheat. Tricin, one of the flavonoid metabolites, was firstly isolated in free form from the rust‐infected emmer wheat (*Triticum dicoccum* L.) leaves (Anderson and Perkin, 1931). Tricin and its derivatives were subsequently believed to exist in a taxonomically limited range of species (Lan *et al.*, 2016). However, tricin conjugates are thought to initiate the lignin formation in several monocot grasses including wheat, starting by incorporating tricin‐(4′‐*O*‐β)‐ethers into the lignin structures (Lan *et al.*, 2015; Lan *et al.*, 2016; Lan *et al.*, 2018). Interestingly, TraesCS1A01G347100 could catalyse the conversion of tricin to tricin 4′‐*O*‐glucoside (Figure 3e), which may inhibit the generation of tricin‐(4′‐*O*‐β)‐ethers. Such a diversion of tricin into lignin renders it important to conduct further investigation of the effect of variation of *TraesCS1A01G347100* on lignification‐related traits such as wheat stem lodging (Zhang *et al.*, 2016; Zheng *et al.*, 2017) or response to biotic stresses (Yang *et al.*, 2017). Hence, focusing on tricin 4′‐*O*‐ decorates may lead to the dissection of wheat‐specific metabolic pathways that take part in the lignification processes and are thereby involved in lignin‐associated agronomic traits.

It has been well known that enzymes responsible for flavonoid glycosylation usually have relatively poor substrate specificities (i.e. they could recognize multiple flavonoids as substrates), and glycosylation of multiple hydroxy groups may be expected (Hong *et al.*, 2007; Isayenkova *et al.*, 2006; Ko *et al.*, 2006; Ko *et al.*, 2008). Our results confirmed with the general rule (poor substrate specificity and multiple glycosylation products) but differed with regard to the detailed glycosylation patterns (Figure S9). Such differences may help us to discover how and why flavonoids are specifically decorated in wheat, especially when the flavonoid decoration processes have been demonstrated to interact with other plant growth events. For instance, starch synthase activity is inhibited by glycosidic flavonoids, which resulted in slower seed setting rates of rice (Zhan *et al.*, 2017), and differentially glycosylated flavonoids were associated with varied UV tolerance in rice (Peng *et al.*, 2017). Moreover, flavonoid metabolites possess high bioactivities when provided in diet (Martin and Li, 2017; Zhou and Ibrahim, 2009), and their contents are associated with food quality and flavour (Sharma *et al.*, 2016; Tieman *et al.*, 2017). Taken together, these features render further flavonoid pathway dissection of key importance in understanding the complex physiological or agronomical traits of wheat.

This is reflected in comparisons between the current mGWAS hits and a recently conducted wheat kernel trait GWAS output (Chen *et al.*, 2019). In the mentioned GWAS study (Chen *et al.*, 2019), both the grain protein content (GPC) and wet gluten content (WGC) traits were associated with the SNP *RAC875_c3187_873* located at 9.88 Mb on chromosome 2D. Coincidently, two amino acids, isoleucine (mr1326) and asparagine (mr1438), were associated with an adjacent SNP *Kukri_c30847_344* residing at 8.30 Mb on chromosome 2D (Table S4), implying that these two amino acids could represent important biomarkers for GPC or WGC. Similarly, flour redness (Fa) mapped by multiple SNPs distributed between 3.30 Mb and 3.39 Mb on chromosome 7D, and flour yellowness (Fb) associated with the *Excalibur_rep_c92684_578* SNP (located at 3.39 Mb on chromosome 7D) are co‐localized with the QTL for metabolite PS068208 (identified as vitamin C) to SNP *tplb0027d07_1388* (4.27 Mb on chromosome 7D, Table S4). This co‐localization is quite persuasive since the varied contents of vitamin C (also known as ascorbic acid) in wheat kernels appear to be an efficient metabolic index for wheat flour whiteness (Junqueira *et al.*, 2008; Niu *et al.*, 2014). These examples illustrate that metabolites could potentially act as biomarkers for respective traits. One benefit to establish this connection is that the subsequent assignment and validation of candidates that affecting agronomic traits may be considerably more straightforward. Another impact would be the simplified working load, which was exemplified by discerning the mysterious root–fungi symbiosis status through detecting the blumenol metabolites in leaves (Wang *et al.*, 2018). It is important to mention, however, that a large proportion of the wheat kernel GWAS hits (Chen *et al.*, 2019) did not co‐localize with our mGWAS hits. This is most likely owing to the fact that these GWAS‐linked SNPs (for instance, *RAC875_c3187_873* that associated with GPC and WPC and *Kukri_c65663_642*, *RAC875_c14064_308*, *Excalibur_c8883_214* and *RAC875_c61016_73* that linked to Fa) were considered to be non‐polymorphic across our wheat population (Table S3). Hence, a parallel design is preferable for the proposed metabolic‐associated agronomic trait investigation.

Collectively, we have presented a comprehensive metabolomic profiling data in wheat kernels and primarily disclosed a flavonoid decoration pathway. Follow‐up exploration of this pathway should include probing the interactions of tricin 4′‐*O*‐ decorates with wheat lignin formation and lignin‐associated agronomic traits. Similarly, more metabolite content–agronomic trait connections may be discovered when focusing on other metabolite groups (Figures S4 and S5). These potential connections include, but are not limited to, the auxin‐responsive traits with the tryptophan metabolism pathway (Mano and Nemoto, 2012; Zhao, 2012) and amino acids as nutrients in wheat (Peng *et al.*, 2018). Moreover, reverse genetic toolboxes (e.g. transgenic approaches) could be applied to investigate the high‐confidence candidate genes (Table 1 and Table S4). For instance, *TraesCS1A01G347100* has been postulated to affect the wheat lignin formation, and investigation on this gene may represent a good lead to alter lignin‐related agronomic traits (Yang *et al.*, 2017; Zhang *et al.*, 2016; Zheng *et al.*, 2017). Similarly, the homoeologous genes *TraesCS4B01G371700* and *TraesCS4D01G365800* were believed to encode sugar transporters (Paulsen *et al.*, 2019), and the target metabolite, sucrose, was linked to wheat yield (Weichert *et al.*, 2017). To this end, our data not only provide specific candidate genes as molecular resources to be utilized after validation, but also enlighten exploration of additional metabolite networks. Such efforts will surely greatly facilitate the metabolomics‐associated breeding of wheat in the future.

## Experimental procedures

### Plant materials

A total of 182 *Triticum* *aestivum* L. accessions were planted in three different environments: Hebei Province (Gaoyi, E 37°62′, N 114°58′) in the 2016–2017 cropping season and Shandong province (Dezhou, E 37°45′, N 116°29′) in the 2016–2017 and 2017–2018 cropping seasons. The detailed information for these wheat accessions, along with how they were planted, managed and harvested, was presented previously (Peng *et al.*, 2018).

### Metabolic profiling

Mature seeds collected from each of the three biological replicates were pulverized using the TissueLyser II machine (Qiagen, Germany) at 29 Hz for 1 min. The metabolites were extracted by adding 1000 μL of 70% methanol (v/v, with pre‐added acyclovir at final concentration of 0.1 mg/L as inner control) to 0.1 g of powder; then, the mixture was vortexed, every 10 min, three times before being kept at 4 °C overnight. The supernatant was generated by centrifuging the re‐vortexed mixture at 8000 ***g*** for 5 min followed by filtering (0.22 μm pore size; ANPEL, Shanghai, China) and was subsequently analysed using an LC–electrospray ionization (ESI)–MS/MS system as previously described (Chen *et al.*, 2013). Briefly, the MS2T library was established using the equant mixture from 50 extractions that were randomly selected from the 182 accessions. The detailed information that contained in the detected metabolite signals (such as the mass‐to‐charge ratio, fragmentation pattern and retention time) was compared with available commercial standards and with metabolite databases (e.g. METLIN, HMDB and MassBank) to facilitate the annotation of metabolites from wheat kernels. Subsequently, the relative contents of each of these 805 identified metabolites (Tables S1 and S2) were quantified using the scheduled multiple reaction monitoring (sMRM) method described previously (Chen *et al.*, 2014). The sMRM algorithm was used with an MRM detection window of 90 s and a target scan time of 1.0 s using Analyst 1.5 software. Given that biological variance is considerably higher than technical variance, we chose not to carry out technical replication.

### Statistical analysis

The broad‐sense heritability was estimated to be *H* ^2^ = Var_G_/(Var_G_ + Var_E_), in which Var_G_ and Var_E_ are for variations of genotype and environment, respectively (Visscher *et al.*, 2008). Linkage disequilibrium (LD) was estimated using standardized disequilibrium coefficients (D′) and squared allele‐frequency correlations (*r* ^2^) for pairs of SNP loci using PopLDdecay software (Zhang *et al.*, 2019), with parameters of MAF = 0.05 and MaxDist = 50Mb. LD plots were generated in Haploview (Barrett, 2009) version 4.2 under default parameters, wherein the *r* ^2^ values were indicated as percentages and displayed as white (*r* ^2^ = 0%) shaded to black (*r* ^2^ = 100%) colours. Relative contents of metabolites were log_2_‐transformed to fit the normal distribution. The hierarchical clustering analysis (HCA) and the heatmaps were obtained through R software version 3.5.1, and the network construction was achieved using version 3.7.1 of Cytoscape software (Shannon *et al.*, 2003) on the basis of metabolites having strong correlation (Spearman's correlation coefficients, *P* < 0.001) to one another. Relative expression levels of candidate genes in wheat grains were retrieved from previously published data (Borrill *et al.*, 2016; Ramirez‐Gonzalez *et al.*, 2018), using the RefSeq 1.0 data set.

### Metabolite genome‐wide association study (mGWAS)

After genotyping the 182 wheat accessions as previously described (Peng *et al.*, 2018), a total of 14 646 polymorphic markers were retained from the 90K SNP array (Wang *et al.*, 2014). The physical positions of these markers on the wheat reference genome (IWGSC, 2018) were utilized to calculate the LD for each chromosome and were employed to investigate the associations between metabolite contents and SNPs. The mGWAS was implemented by a linear mixed model (LMM) using the FaST‐LMM program that provides high‐confidence output and acceptable scan speed (Lippert *et al.*, 2011; Listgarten *et al.*, 2013; Listgarten *et al.*, 2012; Widmer *et al.*, 2014), at thresholds of *P* = 1/14 646 = 6.83 × 10^′5^ after Bonferroni correction, as carried out in previous studies (Guo *et al.*, 2017; Peng *et al.*, 2016). Among the three biological replicates, mGWAS hits appearing at least twice were retained and subjected to subsequent analysis and functional annotation.

### Natural variation of candidate genes among wheat accessions

Primers used in this study, as listed in Table S7, were designed using the PrimerServer online tool (http://202.194.139.32/PrimerServer/) combined with the oligo 7 software (Rychlik, 2007) version 7.60 set at default parameters. PCR amplifications were conducted using the TransStart FastPfu Fly DNA Polymerase or the TransFast Taq DNA Polymerase (TransGen, Beijing, China) following the manufacturer's protocol. Briefly, the PCR conditions were set as follows: after denaturation at 95 °C for 2 min, run 35 cycles of 95 °C for 20 s, Tm for 10 s and 72 °C for varied timing (set as 10 s per 1 kb length of amplicon, see manufacturer's protocol). Finally, the PCR mixtures were kept at 72 °C for 2 min. The single‐band amplicons were directly cloned using the pEASY‐Blunt Zero Cloning Kit (TransGen, Beijing, China), and positive clones from each primer‐sample set were randomly selected and sequenced to acquire the full allelic information of the designated genes from each wheat accession.

### Enzymatic validation of candidate genes

PCR products representing each allelic variation of designated genes were re‐amplified using respective cloned vectors as templates and were directionally cloned into the *pGEX‐6p‐1* expression vector (Novagen) through enzyme digestion and ligation. The primers for cloning the designated genes into expression vectors are listed in Table S7, in which the lower‐cased nucleotides were used for enzyme digestion and subsequent ligation reactions. The error‐free ligates were transformed into the BL21 (DE3) competent cells (TransGen, Beijing, China) and cultured on an LB plate. A single colony was grown in LB media to an OD_600_ value of between 0.6 and 0.8, and recombinant proteins were expressed after induction by adding isopropyl β‐D‐1‐thiogalactopyranoside (IPTG) to a final concentration of 0.1 mm and grown continually for 14 h at 18 °C. Cells were harvested and lysed, and purification of the GST‐tagged proteins was performed using the glutathione Sepharose 4B (GE Healthcare, Chicago, America) following the manufacturer's instructions. The purified proteins were stored at −80 °C until future experiments.

Standard *in vitro* enzyme assays for the role of enzymes encoded by *TraesCS2B01G472300*/*TraesCS2B01G472400* and *TraesCS1A01G347100/TraesCS1A01G347200* were performed in a total of 10 μL reaction mixture containing 3.5 μL purified protein, 2 μL of 1 m Tris‐HCl (pH = 7.4) and 1 μL of 1 mm substrate. In validating the glycosylation activities, 2 μL of 50 mm MgCl_2_ and 0.5 μL of 15 mm UDP‐glucose were added into the reaction mixture, with numerous flavonoids utilized as substrates (e.g. apigenin, tricin, kaempferol, quercetin and 3′, 4′, 5′‐*O*‐trimethyltricetin), whereas the malonylation reaction required 0.3 μL of 1 mm malonyl‐coenzyme A, using flavonoid *O*‐glucoside conjugates as substrates (including apigenin 5‐*O*‐glucoside, apigenin 7‐*O*‐glucoside, apigenin 6‐*C*‐glucoside, apigenin 8‐*C*‐glucoside, eriodictyol 7‐*O*‐glucoside, luteolin 7‐*O*‐glucoside, naringenin 7‐*O*‐glucoside, kaempferol 3‐*O*‐glucoside and tricin 7‐*O*‐glucoside). After incubating at 37 °C for 30 min, the reaction was stopped by adding 30 μL of methanol and used for LC‐MS analysis. Each enzyme assay, as well as the ones that utilize expressed protein from *pGEX‐6p‐1* empty vector as negative controls, was conducted in duplicate.

### Enzyme kinetics

After validating the enzymatic activity for TraesCS2B01G472400 and TraesCS1A01G347100, kinetic constants for them were further determined. In brief, varied substrates (1 μL of 0.2 μm to 500 μm tricin for TraesCS1A01G347100 and 1 μL of 1 μm to 1 mm tricin 7‐*O*‐glucoside for TraesCS2B01G472400) were supplied into the reaction mixture. Kinetic parameters were calculated using a Michaelis–Menten model (Sigma Plot, version 14.0). All the reactions were run in duplicate, and each experiment was repeated twice.

### Phylogenetic analysis

Neighbour‐joining phylogenetic trees were constructed from the alignment of the amino acid sequences of the respective genes, using version 7.0.26 of MEGA software (Kumar *et al.*, 2016) set at default parameters. The bootstrap method, based on 1000 replicates, was applied for the functional annotation of the phylogenetic trees.

## Conflicts of interest

The authors declare that they have no conflicts of interest.

## Author contributions

W.C. and Z.H. conceived the project. W.C., J. L. and J.C. designed the experiments. J.C. conducted the experiments. J.C. and X.H. performed the main data analysis. T.S., Y.H., X.X. and D.S. worked on the field material management. H.Y. carried out the LC‐MS analyses. J.C., W.C. and A.R.F. wrote the article.

## Supporting information


**Figure S1** Distribution of metabolite contents before and after normalization.
**Figure S2** Statistical data of 805 metabolites amongst the 182 wheat accessions.
**Figure S3** Correlation network of metabolites.
**Figure S4** The indole‐ring skeletons contained metabolites shared high correlation with each other.
**Figure S5** The identification of amino acids and nucleotides metabolite groups.
**Figure S6** Sequence alignments of three candidate genes.
**Figure S7** Enzymatic assay of flavonoid glucoside conjugates catalyzed by TraesCS2B01G472400.
**Figure S8** TraesCS1A01G347100 and TraesCS2B01G472400 could sequentially glycosylate and then malonylate flavonoids.
**Figure S9** The flavonoid pathway dissected in the current study.

**Table S1** Detailed information for metabolites detected in the current study.
**Table S2** Wheat accessions and corresponding metabolomic data in the current study.
**Table S3** The 14 646 polymorphic SNP markers information derived from the 90 K chip.
**Table S4** Detailed information for the 1098 mGWAS associations.
**Table S5** SNPs and metabolites information for the top 10 mGWAS hotspots.
**Table S6** Linkage disequilibrium for each chromosome.
**Table S7** Primers used in the current study.
