# Supplementary material for: Metabolite‐based genome‐wide association study enables dissection of the flavonoid decoration pathway of wheat kernels
Source: Plant Biotechnol J. 2020 Mar 21;18(8):1722–35. doi: 10.1111/pbi.13335 (PMC7336285; doi:10.1111/pbi.13335)
Supplement: Supplementary file 1 — Figure S1 Distribution of metabolite contents before and after normalization. Figure S2 Statistical data of 805 metabolites amongst the 182 wheat accessions. Figure S3 Correlation network of metabolites. Figure S4 The indole‐ring skeletons contained metabolites shared high correlation with each other. Figure S5 The identification of amino acids and nucleotides metabolite groups. Figure S6 Sequence alignments of three candidate genes. Figure S7 Enzymatic assay of flavonoid glucoside conjugates catalyzed by TraesCS2B01G472400. Figure S8 TraesCS1A01G347100 and TraesCS2B01G472400 could sequentially glycosylate and then malonylate flavonoids. Figure S9 The flavonoid pathway dissected in the current study. [file PBI-18-1722-s001.pdf]

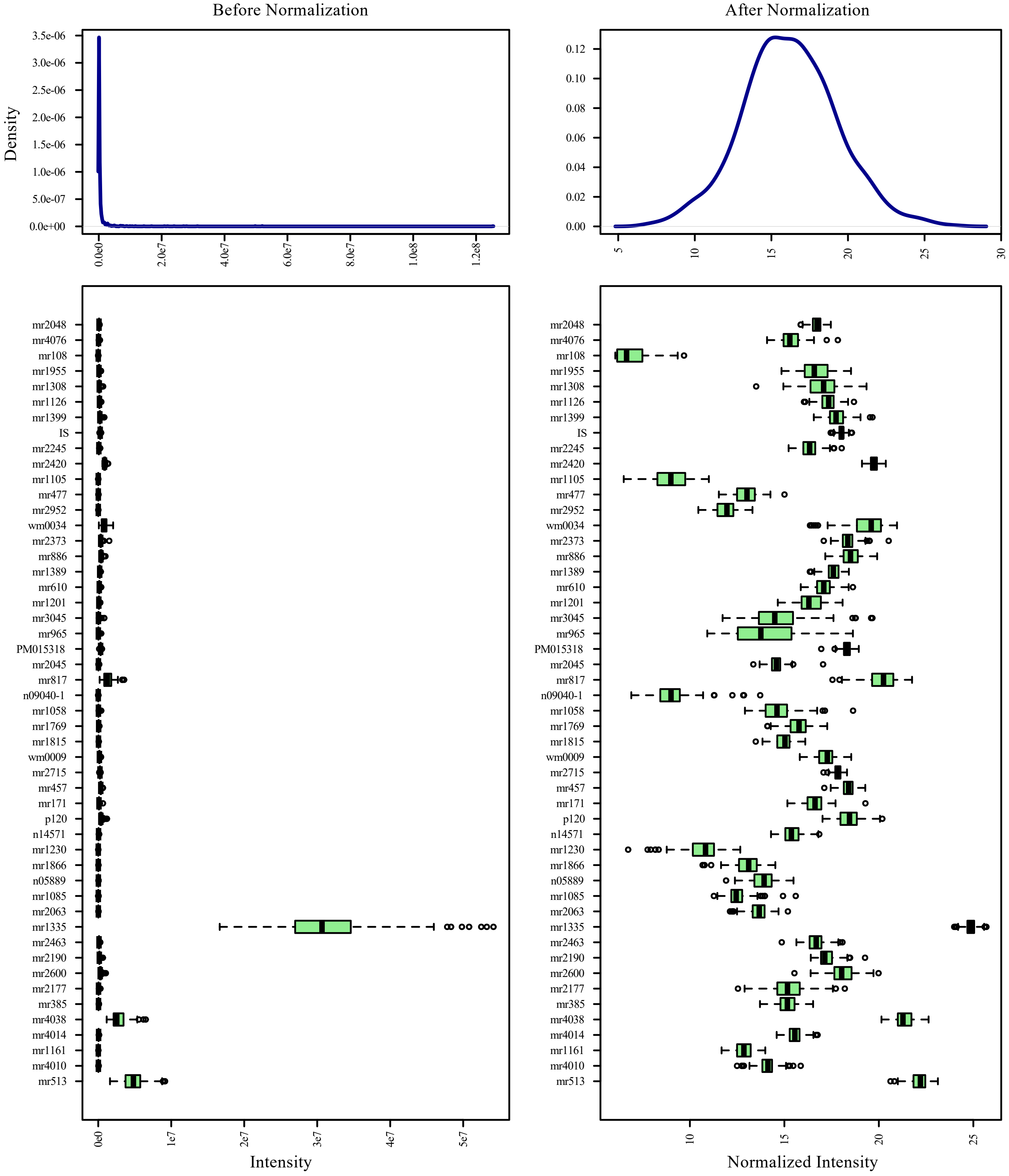

Figure S1 Distribution of metabolite contents before and after normalization.

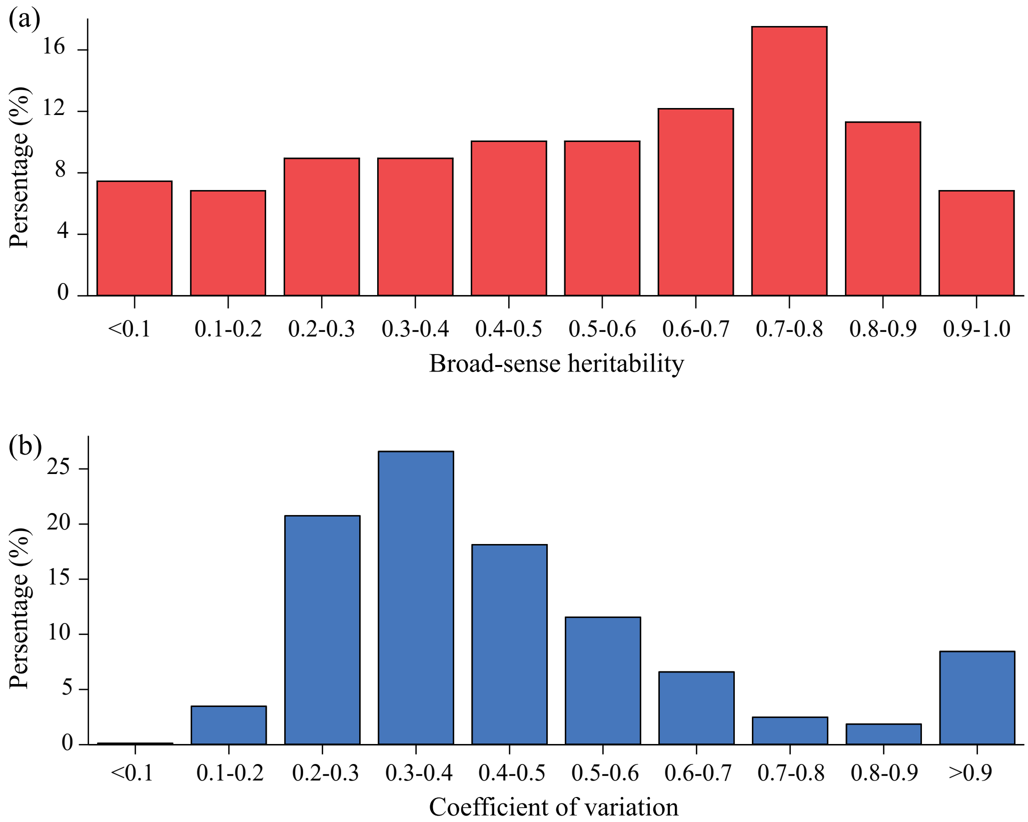

Figure S2 Statistical data of 805 metabolites amongst the 182 wheat accessions. Details for the broad-sense heritability (a) and coefficient of variations (b) of the 805 metabolites.

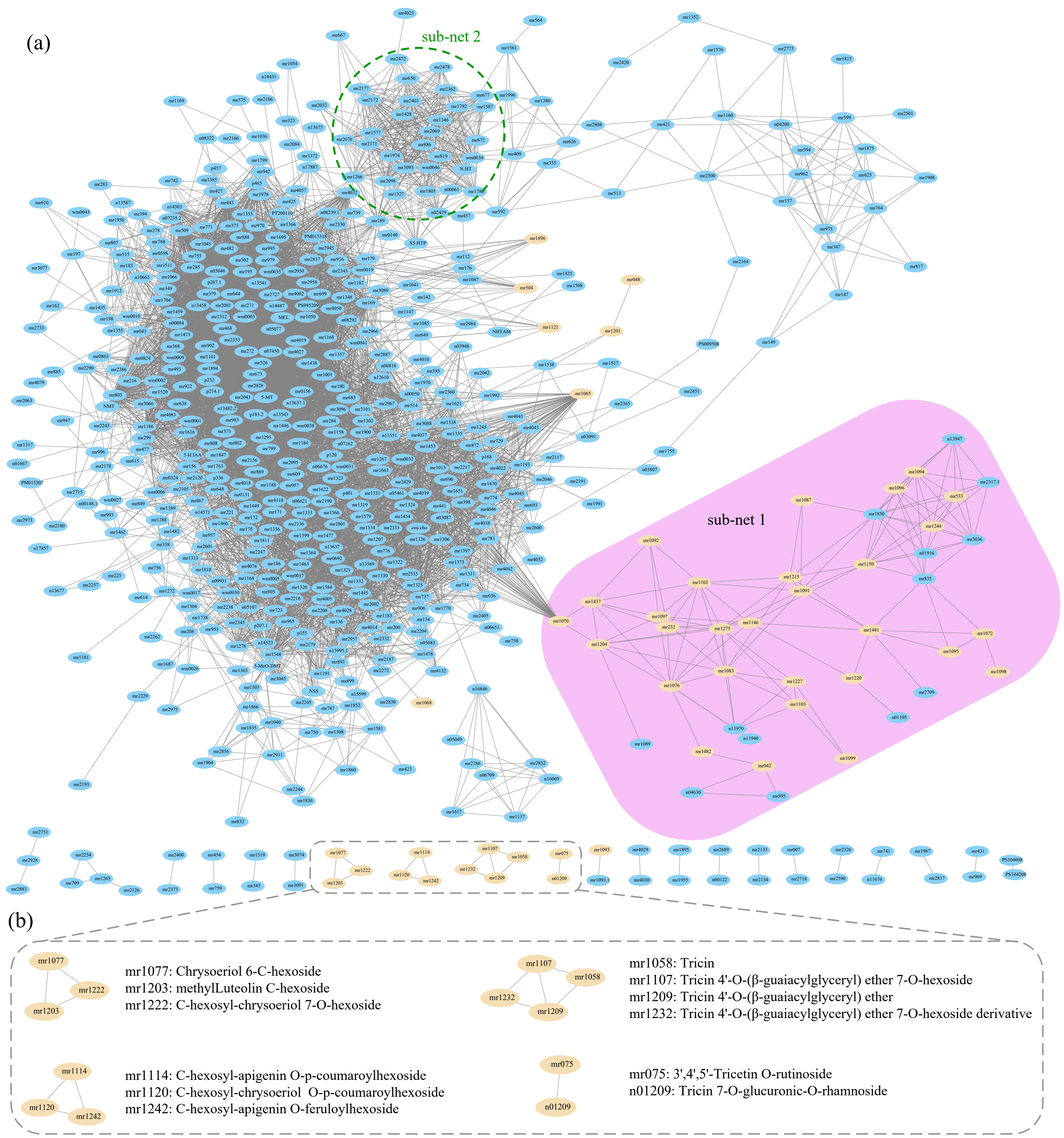

Figure S3 Correlation network of metabolites. This network was constructed under cut-off coefficient index of 0.55, each ellipse represents metabolites with IDs labeled within, and lines connected different metabolites share similar distribution patterns (with Spearman coefficient index over 0.55) amongst 182 wheat accessions. Flavonoid metabolites were displayed by wheat-colored ellipses, and the green-circle included or violet-backgrounded nets were more detailly presented in Figures S4 and 1b, respectively.

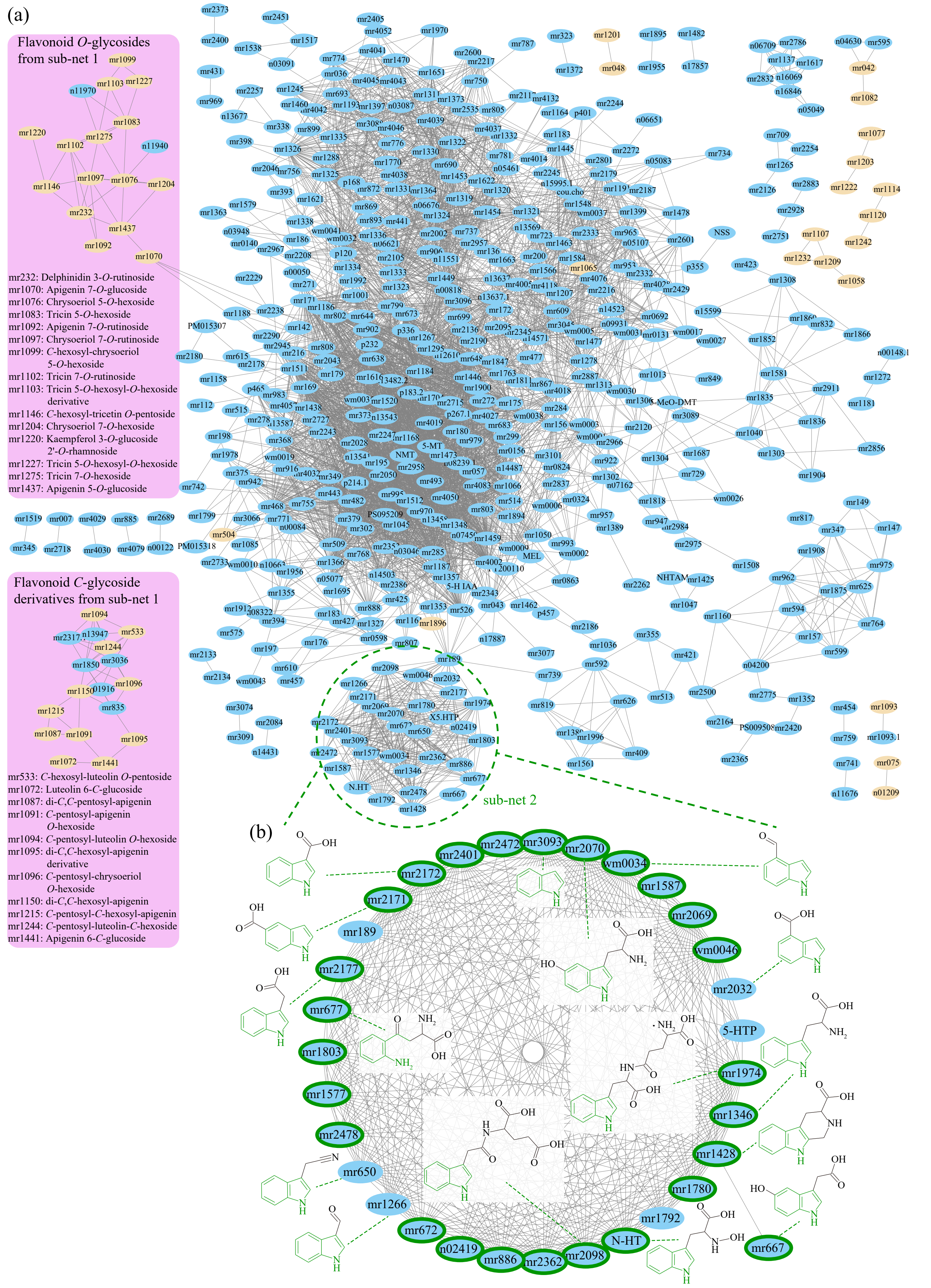

Figure S4 The indole-ring skeletons contained metabolites shared high correlation with each other. (a) This network was obtained at coefficient value of 0.60, in which the flavonoid glycosides were separated as postulated in Figure 1b, and the green-circled sub-net in Figure S3a was more easily discerned. (b) The green-circled network may represent tryptophan metabolism. The common indole-ring skeletons are displayed as green within the chemical structures for each of the currently identified metabolites, and the green-ringed IDs corresponded to the green-rectangles including metabolites in Figure 1a.

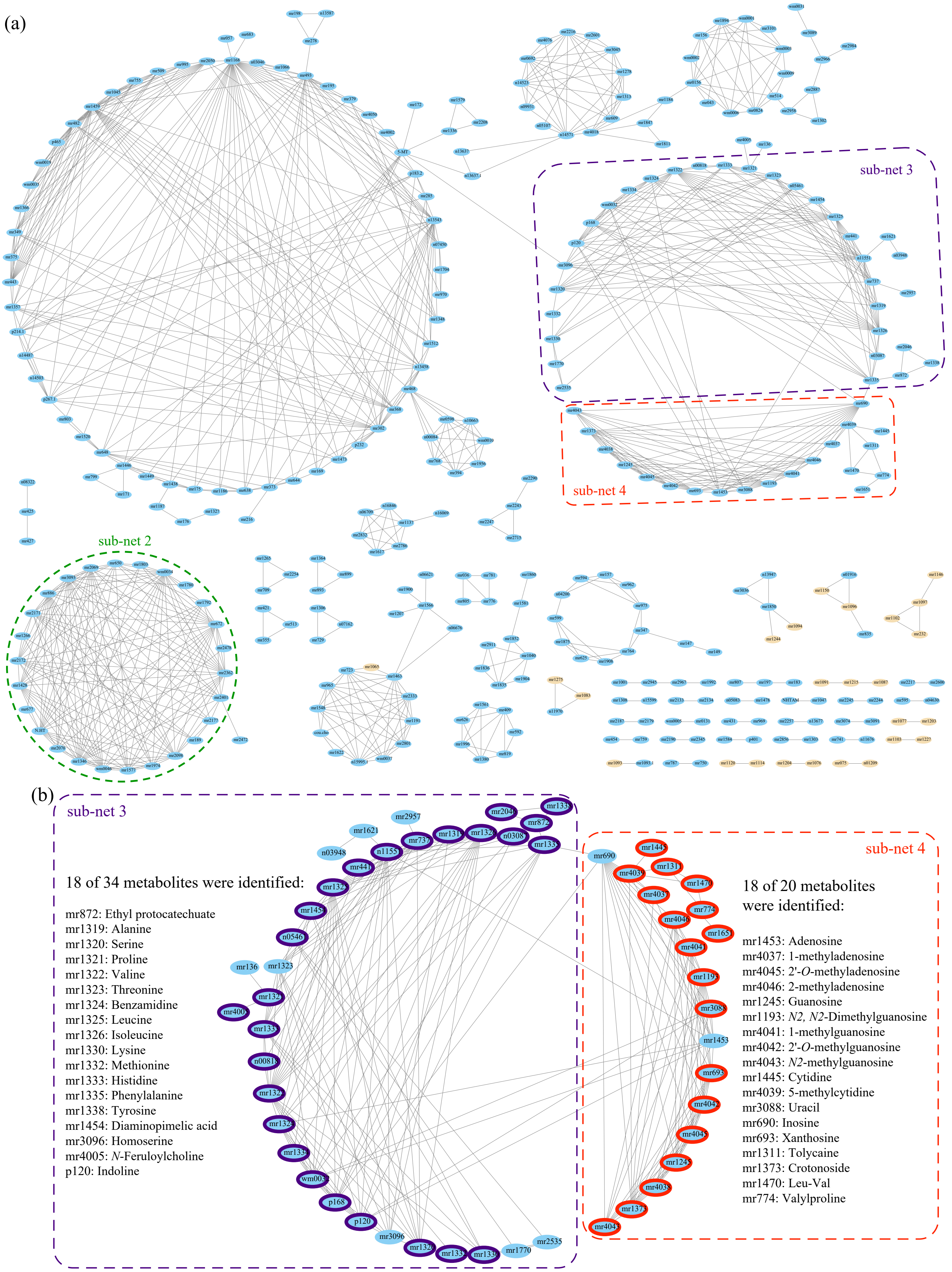

Figure S5 The identification of amino acids and nucleotides metabolite groups. (a) The coefficient cut-off value was set as 0.75, and the amino acids (sub-net 3 in indigo) or nucleotides (sub-net 4 in scarlet) were separated. The tryptophan metabolism network was presented (sub-net 2 in green), whilst much fewer flavonoid metabolites (wheat-colored cells) were included. (b) Detailed information for the amino acid and nucleotide sub-nets, in which the indigo- or scarlet-color ringed IDs were included in the respective colored rectangles in Figure 1a, and names for the identified metabolites were respectively listed.

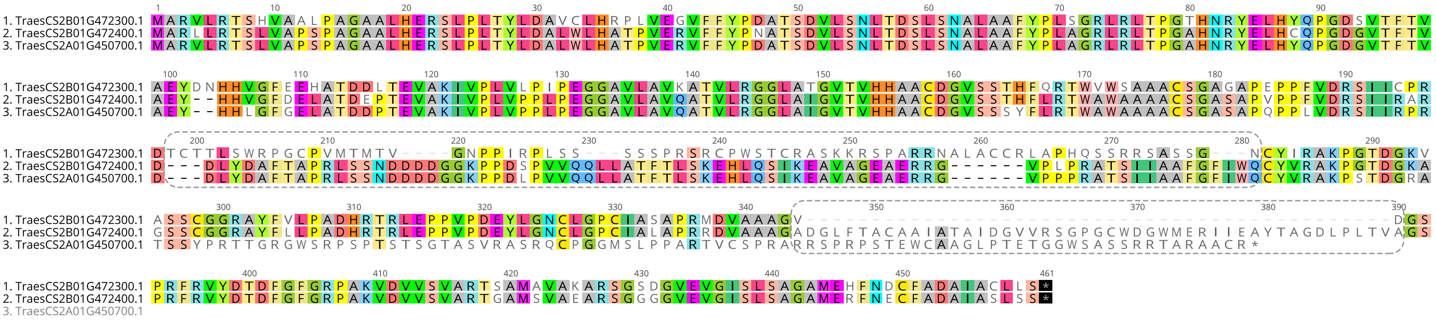

Figure S6 Sequence alignments of three candidate genes. The grey dashed boxes denote major sequence variations among the three amino acid sequences.

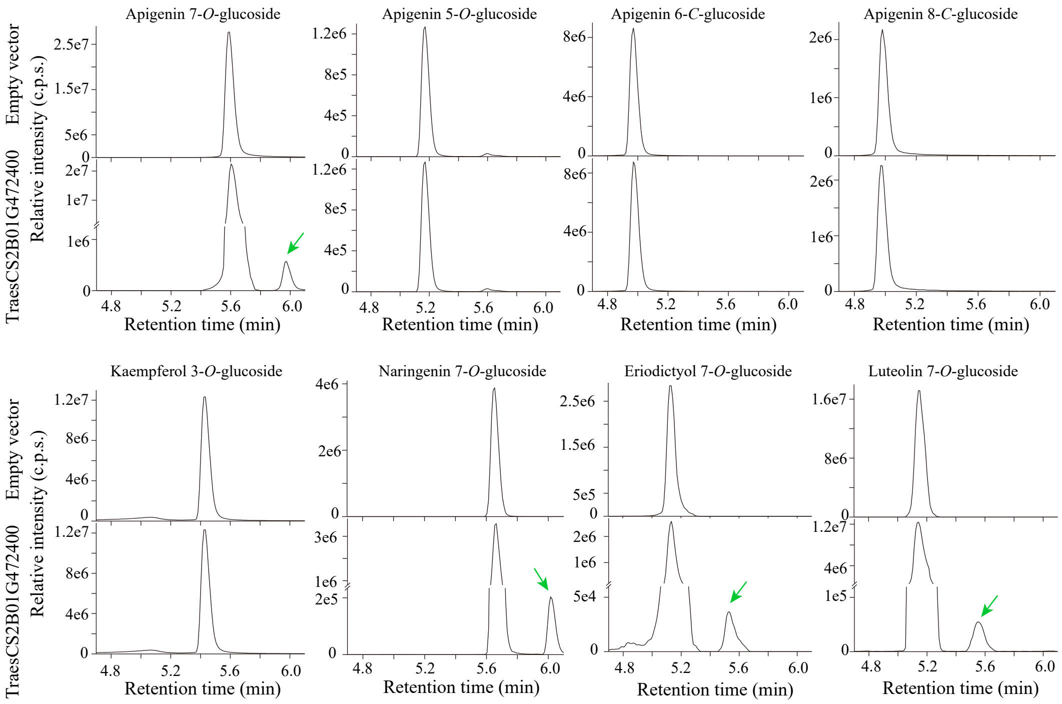

Figure S7 Enzymatic assay of flavonoid glucoside conjugates catalyzed by TraesCS2B01G472400. Enzymatic assays were conducted using eight flavonoid glucosides as substrates, and the arrows pointed to the expected products. MS spectrum for these detected product peaks were displayed in Figure 5.

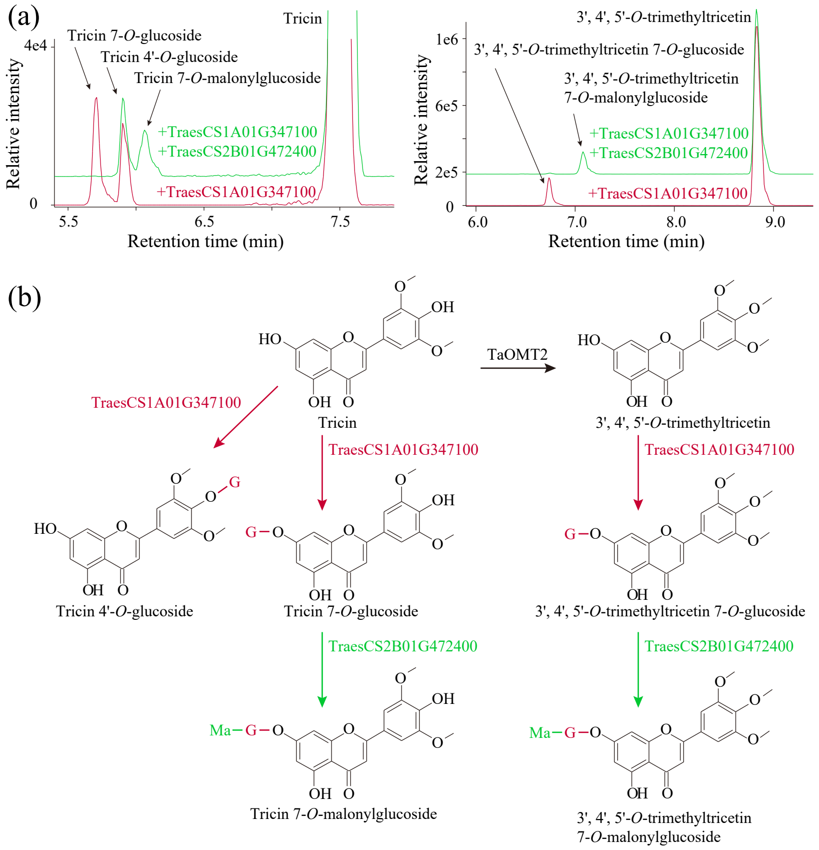

Figure S8 TraesCS1A01G347100 and TraesCS2B01G472400 could sequentially glycosylate and then malonylate flavonoids. (a) Enzymatic products detected when adding single (TraesCS1A01G347100) or both enzymes. (b) Summary of catalytic reactions for these two enzymes.

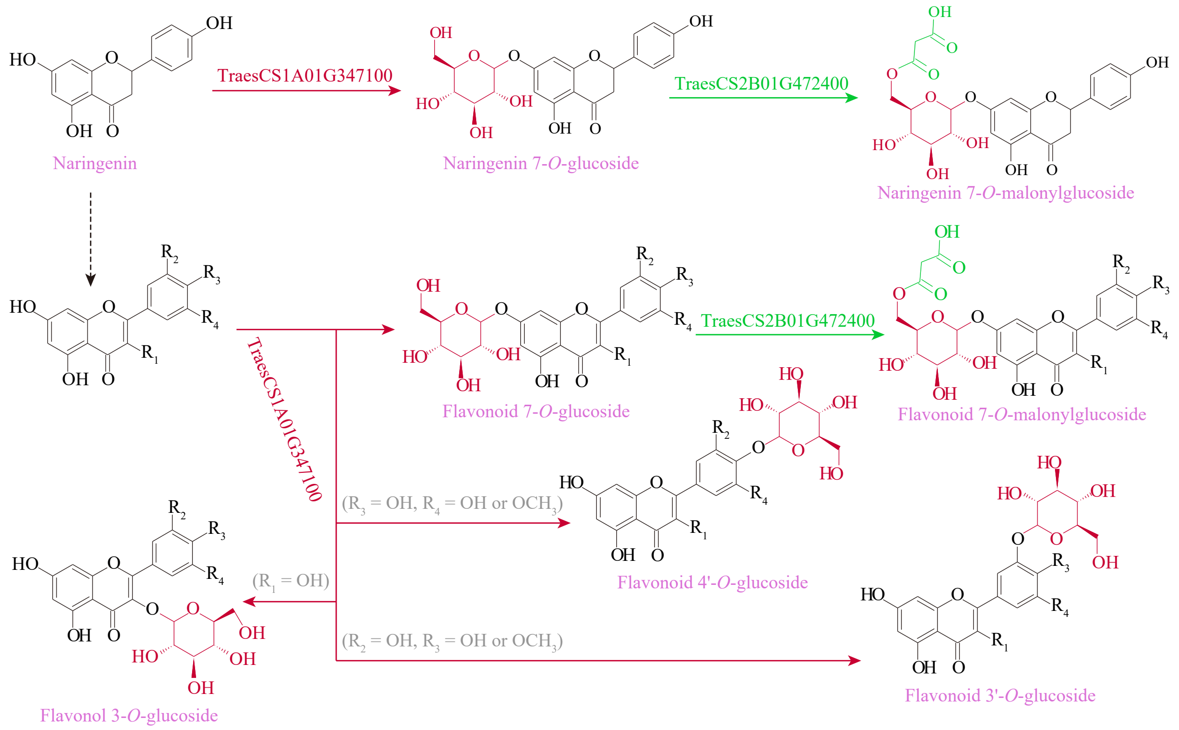

Figure S9 The flavonoid pathway dissected in the current study. Reactions catalyzed by TraesCS1A01G347100 and TraesCS2B01G472400 are labeled by red and green colors, respectively. The chemical names were indicated at the bottom of each structure in violet, and the minimum structural requirement for each reaction are presented in grey text upon respective arrows.
